# Diltiazem efficacy and CYP2D6 gene polymorphism in patients with atrial fibrillation with rapid ventricular response

**DOI:** 10.1186/s43044-023-00375-0

**Published:** 2023-06-16

**Authors:** Mehmet Uluturk, Atakan Yilmaz, Murat Seyit, Mert Ozen, Alten Oskay, Aykut Kemanci, Medine Unal, Hande Senol, Aylin Koseler, Ibrahim Turkcuer

**Affiliations:** 1 Department of Emergency Medicine, Faculty of Medicine, Pamukkale University, 20070 Denizli, Turkey; 2 Department of Biostatistics, Faculty of Medicine, Pamukkale University, 20070 Denizli, Turkey; 3 Department of Biophysics, Faculty of Medicine, Pamukkale University, 20070 Denizli, Turkey

**Keywords:** AF with RVR, CYP2D6, Diltiazem, Genetic polymorphism

## Abstract

**Background:**

Diltiazem stands out as one of the front-line drugs administered in the emergency department to achieve acute rate control in patients suffering from atrial fibrillation with rapid Ventricular Response. One of the cytochrome enzymes involved in the metabolism of diltiazem is cytochrome P450 2D6 (CYP2D6). Interindividual differences can act on drug metabolism and thus drug efficacy due to the genetic polymorphism induced by the CYP2D6 enzyme. This study explores the association between the efficacy of diltiazem and the genetic polymorphism of CYP2D6 in patients with atrial fibrillation with rapid ventricular response.

**Results:**

87 out of 93 individuals with ventricular rate > 120 beats/min constituted the patient cohort. The patients were administered 0.25 mg/kg diltiazem intravenously. As a second dose, 0.35 mg/kg diltiazem was administered to patients who reportedly did not receive adequate drug efficacy. Heart rate control was considered to be achieved in patients whose heart rate fell below 110 beats/min and did not rise above 110 beats/min for 2 h. CYP2D6 *2, *3, *4 and *10 represent allele variants and *1 represents wild type (wt) allele. Achieving rate control after one or two doses of diltiazem in normal allele (wt/wt) carriers proved significantly higher than wt/*2, wt/*4 and wt/*10 heterozygous variant carriers. No significant difference was noted in wt/*3 heterozygous variant carriers.

**Conclusion:**

The presence of *2, *4 and *10 alleles was observed to significantly compromise the drug efficacy. *3 allele was found to bear no relation to the effect of diltiazem on achieving rate control.

## Background

Atrial fibrillation (AF) can be defined as high-frequency stimulation of the atrium. As a result of this high-frequency stimulation, atrial contractions that are not synchronised with each other develop and as a result, the ventricles are stimulated irregularly [1]. In *Framingham Heart Study*, William Kannell et al. [2] designated AF as a major contributor to cardiac and cerebrovascular mortality, highlighting its epidemiological importance for the first time. Growing progressively more prevalent with the aging populations across the world, AF has now become the most common arrhythmia type.

Specific drugs, especially non-dihydropyridine-derived calcium channel blockers, such as verapamil and diltiazem and beta-blockers are administered to achieve acute rate control for patients admitted into the ED for a range of reasons and diagnosed with AF with rapid ventricular response (RVR) after heart rate monitoring or electrocardiographic evaluation. Diltiazem is one of the first-line drugs preferred for acute treatment in EDs due to its enhanced anti-hypertensive and retarding efficacy. However, it is occasionally observed that the rate control cannot be adequately achieved in diltiazem administration, so physicians either have to provide repeated doses or resort to another retarding agent. Such a scenario exposes patients to repeated dosing, heightening the risk of side effects. Moreover, follow-up and treatment duration in ED are prolonged since certain period of time is required during repeated dosing and administration of drug doses.

Drug elimination is largely dependent on metabolic changes mediated by enzymes. Changes in genetic structure may cause abnormal changes in the pharmacokinetics of drugs in some individuals. The main reason behind the variation of drug elimination according to genetic structure is the genetic polymorphism of the synthesis rate and/or quality of the enzymes involved in drug metabolism. If the enzyme polymorphism is in the form of deficiency or inactivity of the gene that forms the enzyme, drug metabolism through the enzyme does not occur. If the enzyme is partially synthesised in a reduced form, the rate of metabolism is reduced. Another situation is when the genetic polymorphism causes a functional defect in the synthesised enzyme without affecting the rate of synthesis; in this case, the substrate specificity of the drug is altered [3].

Cytochrome enzymes in the liver are primarily involved in the metabolizing of diltiazem and many similar drugs. Drug metabolism rate, drug efficacy, and side-effect profile might vary as a result of genetic polymorphism which may impair the functions of cytochrome enzymes and change the specificity of the enzyme to the substrate. One of the cytochrome enzymes involved in the metabolism of diltiazem is cytochrome P450 2D6 (CYP2D6). Interindividual differences can act on drug metabolism and thus drug efficacy due to the genetic polymorphism of the CYP2D6 enzyme. CYP2D6 is highly polymorphic. Although CYP2D6 has more than 100 known variant alleles, more than 95% of phenotyping can be done with only 9 alleles. *1 and *2 alleles are fully functional, *10, *17 and *41 alleles are associated with decreased function, *3, *4, *5 and *6 are not functional [4, 5].

This study set out to investigate whether the identification of gene profiles can guide the retarding treatment in the acute period by analyzing the association between the efficacy of diltiazem and the genetic polymorphism of CYP2D6 in patients admitted into ED for various reasons and diagnosed with AF with RVR after electrocardiographic examination of their heart rhythms.

## Methods

### Study type

This prospective study elucidates the relationship between the effectiveness of diltiazem administered as a retarding treatment and the CYP2D6 gene polymorphism in patients presenting to ED for various reasons and diagnosed with AF with RVR after their electrocardiographic examination.

Prior to the study, the ethics approval was granted by Non-Interventional Clinical Research Ethics Committee of Pamukkale University (date: July 28, 2020, number: 14). In accordance with the Declaration of Helsinki, written informed consent was obtained from all the participants enrolled in the study. This research supported by the grant from Pamukkale University, Scientific Research Projects Fund (2021TIPF001).

### Study population

**Fig. 1:**
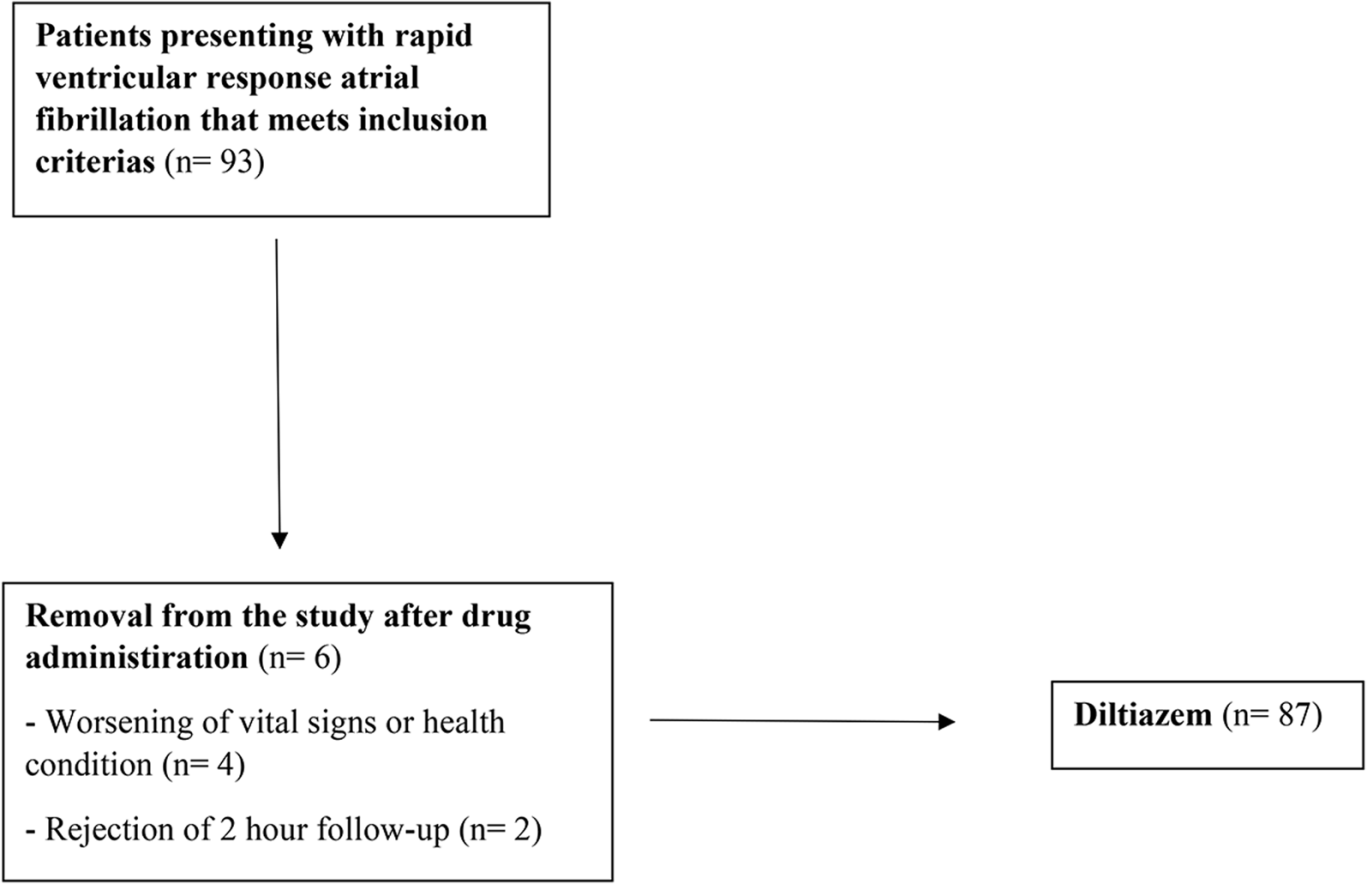
Study design chart

Carried out between June 1, 2020 and October 1, 2021 at the ED of a tertiary hospital with a capacity of approximately 130,000 patients per year, this clinical trial was primarily managed by a research assistant and faculty member for 24 h. 87 out of 93 individuals with ventricular rate > 120 beats/min constituted the patient cohort, while the control cohort consisted of 100 healthy volunteers. The physical examinations of the volunteers in the control cohort were evaluated and all of them were healthy. 6 volunteers from the patient cohort were removed from the study after drug administration due to worsening vital signs and health conditions or rejection of 2 h follow-up (Fig. 1).

### Subject selection

#### Inclusion criteria for the patient cohort

1. Having a ventricular rate above 120 beats/min due to any reason and being diagnosed with AF with RVR after their electrocardiographic examination.
2. Having a systolic blood pressure of 90 mmHg and over or a mean arterial pressure (MAP) above 65 mmHg.
3. Being aged 18 and over and volunteering to participate in the study.

#### Inclusion criteria for the control cohort

1. Being aged 18 and over and being healthy.

#### Exclusion criteria for the patient cohort

1. Systolic blood pressure < 90 mmHg or mean arterial blood pressure < 65 mmHg on admission.
2. History of severe heart failure (NYHA 3 and 4).
3. Left ventricular ejection fraction < 40%.
4. Acute myocardial infarction suffered in the last one month.
5. Preexcitation syndrome or QRS width > 0.12 s on electrocardiography.
6. Septic or cardiogenic shock.
7. Conditions causing compensatory tachycardia such as fever, tachypnea (> 25/min), and low saturation (sO2 < 92).
8. Allergy to diltiazem.
9. Intake of any anti-arrhythmic medication such as diltiazem, beta blocker, and digoxin prior to hospital admission.
10. Liver failure.
11. Refusal to participate in the study.

### Research protocol

#### Genotyping

A 2-ml blood sample was taken in vacutainer tubes containing ethylenediamine tetra-acetic acid (EDTA) and kept at a frozen state at − 80 °C until the analysis. Genomic DNA isolation was performed through the standard phenol chloroform method. CYP2D6 *2, *3, *4 and *10 represent allele variants and *1 represents wild type (wt) allele. The genotype analyses for the CYP2D6 *2, *3, *4 and *10 alleles were performed on PCR by sequencing the ABI PRISM 7700 Sequence System (USA). The primers used for PCR and DNA sequencing were as follows:

For CYP2D6*2 polymorphism; 5′-CTGACAGGTGCAGAATTGGAG-3′ and 5′-CATCCCGGCAGAGAACAG-3′ primers,

For CYP2D6*3 polymorphism; 5′-GATGAGCTGCTA ACTGAGCTC-3′ and 5′-GCCTCCCCTCATTCCTCCT-3′ primers,

For CYP2D6*4 polymorphism; 5′-GTGGGTGATGGGCAGAAG-3′ and 5′-GAGGGAGGCGATCACGTT primers,

For CYP2D6*10 polymorphism, CAACGCTGGGCTGCACGgT-3′ and 5′-GCCCTTGCCCTACTCTTCCTTGG-3′ [6].

### Drug dosage and administration

#### Diltiazem (25 mg) (Diltizem-L^®^, Mustafa Nevzat, Istanbul, Turkey); 25 mg/5 mL

The patient cohort was administered diltiazem 0.25 mg/kg in the IV form. As a second dose, diltiazem 0.35 mg/kg was administered to patients who reportedly did not receive adequate drug efficacy. 15-min intervals were left between the doses to observe drug efficacy. During this period, the vital parameters were noted as baseline (0 h), 1 h, and 2 h after the treatment. Heart rate control was considered to be achieved in the patients whose heart rate fell below 110 beats/min and did not rise above 110 beats/min for 2 h. Those developing side effects (bradycardia, SBP < 90 mmHg, MAP < 65 mmHg, allergy) in the treatment process were excluded from the study.

The demographic characteristics of the patients such as gender and age as well as their hemato-biochemical parameters parameters (BUN, urea, creatinine, AST, ALT, CRP, Na, K, etc.) were computerized, and the relevant statistical analyses were performed.

### Data collection

The electrocardiographs were performed with GE Healthcare Mac 2000® for electrocardiographic evaluation of the patients and then computerized into the digital environment. Bedside cardiac echocardiography was performed with Mindray M7 Premium® ultrasound device to identify their baseline left ventricular ejection fraction values. A GE B40® screen was used to monitor their vital parameters. In addition, the required information such as age, gender, history of chronic diseases, AF history, and drug regimen were recorded. The treatment phase was followed up by a healthcare provider.

### Power & statistical analysis

Our power analysis calculated that, when 164 individuals were recruited in the study (82 patients, 82 healthy controls), an effect size of 0.80 with an alpha level of 0.05 would be achieved, with the prediction of 20% polymorphism in the patients with diltiazem treatment and 5% in the controls.

All the statistical data were analyzed with SPSS v.25 package program (IBM SPSS Statistics 25 software (Armonk, NY: IBM Corp.)). Conformity of continuous variables to normal distribution was calculated by the Kolmogorov–Smirnov test. The descriptive statistics were expressed as frequency (n) and percentage (%) for categorical variables, and as mean, standard deviation, median, minimum, and maximum values for numerical variables. Pearson Chi-Square and Fisher's Exact tests were run to analyze categorical variables. As far as the analyses of more than two measurements are concerned, the Repeated Measures variance analysis (post hoc: Bonferroni) was performed for the comparison of parametric continuous variables, while the Friedman Test (post hoc: Wilcoxon paired two-sided test with Bonferroni correction) was used to compare non-parametric continuous variables. The cases with an alpha level below 0.05 were considered statistically significant.

## Results

The mean age of the patients (n = 87) and controls (n = 100) was 69.3 ± 14.1 and 57.1 ± 16.1 years, respectively (*p* = 0.001). 56.3% of the patients and 46% of the controls consisted of female subjects (*p* = 0.159).

Blood pressure and pulse rate were measured at 0 h before the diltiazem administration and at 1 h and 2 h after the administration. The difference between the baseline (0 h) and 1 h and 2 h systolic blood pressure measurements was statistically significant (Δ_1_ = 12.08 ± 19.51, *p* = 0.0001; Δ_2_ = 13.56 ± 21.46, *p* = 0.0001). However, no significant change occurred between the 1 h and 2 h. (Δ_3_ = 1.48 ± 17.19, *p* = 1,000). A significant difference was noted between 0 and 2 h diastolic blood pressure measurements of the patients (Δ_5_ = 7.49 ± 18.42, *p* = 0.006). The changes in the pulse rate values of the patients at 0–1 h, 0–2 h, and 1–2 h turned out to be significant in all three comparisons (Δ_7_ = 35.71 ± 25.04, *p* = 0.0001; Δ_8_ = 42.05 ± 25.84, *p* = 0.0001; Δ_9_ = 6.33 ± 23.09, *p* = 0.0001) (Table 1).

**Table 1:** Systolic blood pressure, diastolic blood pressure, and heart rate measurements as well as 0 h, 1 h and 2 h measurements, and their statistical association

Systolic blood pressure (SBP)	0 h SBP	1 h SBP	Δ_1_ = 12.08 ± 19.51 p* = 0.0001
Mean ± S.D	137.39 ± 25.95	125.31 ± 21.67	
	0 h SBP	2 h SBP	Δ_2_ = 13.56 ± 21.46 p* = 0.0001
Mean ± S.D	137.39 ± 25.95	123.83 ± 18.78	
	1 h SBP	2 h SBP	Δ_3_ = 1.48 ± 17.19 p* = 1.000
Mean ± S.D	125.31 ± 21.67	123.83 ± 18.78	

Diastolic blood pressure (DBP)	0 h DBP	1 h DBP	Δ_4_ = 5.07 ± 19.3 p* = 1.000
Mean ± S.D	83.44 ± 17.85	78.37 ± 16.27	
	0 h DBP	2 h DBP	Δ_5_ = 7.49 ± 18.42 p* = 0.006
Mean ± S.D	83.44 ± 17.85	75.94 ± 14.8	
	1 h DBP	2 h DBP	Δ_6_ = 2.43 ± 12.81 p* = 0.092
Mean ± S.D	78.37 ± 16.27	75.94 ± 14.8	

Pulse rate (PR)	0 h PR	1 h PR	Δ_7_ = 35.71 ± 25.04 p** = 0.0001
Mean ± S.D	152.89 ± 19.01	117.17 ± 23.17	
	0 h PR	2 h PR	Δ_8_ = 42.05 ± 25.84 p** = 0.0001
Mean ± S.D	152.89 ± 19.01	110.84 ± 26.52	
	1 h PR	2 h PR	Δ_9_ = 6.33 ± 23.09 p** = 0.0001
Mean ± S.D	117.17 ± 23.17	110.84 ± 26.52	

Bold values indicate *p* < 0.05 which is statistically significant

p* values derived from the Wilcoxon paired two-sided test with Bonferroni correction

p** values derived from the Bonferroni test

When we compared the distribution of polymorphisms of CYP2D6*2, CYP2D6*3, CYP2D6*4 and CYP2D6*10 alleles between patient and control cohorts, there were statistically significant differences between two groups for CYP2D6*2 and CYP2D6*10 alleles (*p* = 0.001 and *p* = 0.0001, respectively) (Table 2).

**Table 2:** The distrubitions of CYP2D6*2, CYP2D6*3, CYP2D6*4 and CYP2D6 *10 alleles in patient and control group

	Patient cohort (%)	Control cohort (%)	Total (%)	
CYP2D6*2				
wt/wt	56 (39.4)	86 (60.6)	142 (100)	***p* = 0.001**
wt/*2	31 (68.9)	14 (31.1)	45 (100)	
*2/*2	0 (0)	0 (0)	0 (0)	
CYP2D6*3				
wt/wt	61 (44.9)	75 (55.1)	136 (100)	*p* = 0.454
wt/*3	26 (51)	25 (49)	51 (100)	
*3/*3	0 (0)	0 (0)	0 (0)	
CYP2D6*4				
wt/wt	62 (42.8)	83 (57.2)	145 (100)	*p* = 0.055
wt/*4	25 (59.5)	17 (40.5)	42 (100)	
*4/*4	0 (0)	0 (0)	0 (0)	
CYP2D6*10				
wt/wt	78 (53.4)	68 (46.6)	146 (100)	***p* = 0.0001**
wt/*10	9 (22)	32 (78)	41(100)	
*10/*10	0 (0)	0 (0)	0 (0)	

Bold values indicate *p* < 0.05 which is statistically significant

p* values derived from Pearson Chi-square test

p** values derived from Fisher's exact chi-square test

Heterozygous *2 allele was established in 31 patients, while homozygous *2 allele was observed in none of the participants. Adequate rate control after one or two doses of diltiazem in the individuals carrying the normal allele (wt/wt) (n = 40; 71.4%) proved significantly higher than those with the wt/*2 heterozygous variant gene (n = 12; 38.7%) (*p* = 0.003). On the other hand, heterozygous *3 allele was detected in 26 patients, whereas homozygous *3 allele was undetectable among our participants. Ensuring successful rate control following the administration of one or two doses of diltiazem in the patients with the normal allele (wt/wt) (n = 39; 63.9%) and in those with the wt/*3 heterozygous variant gene (n = 13; 50%) did not yield a significant difference (*p* = 0.225). In addition, heterozygous *4 allele was found in 25 individuals in the patient cohort, but homozygous *4 allele was unobserved in our study. When those carrying the normal allele (wt/wt) (n = 47; 75.8%) were compared to their counterparts with the wt/*4 heterozygous polymorphic gene (n = 5; 20%), the provision of rate control after diltiazem dosing proved to be significantly higher in the former (*p* = 0.0001). Finally, heterozygous *10 allele was observed in 9 individuals in the patient cohort, but homozygous *10 allele was not detected among our participants. No rate control could be achieved with diltiazem administration in any of the heterozygous *10 allele carriers. Besides, the case of achieving adequate rate control after diltiazem dosing was significantly higher in the individuals with the normal variant allele (wt/wt) (n = 52; 66.7%) than those carrying the wt/*10 heterozygous polymorphic gene (n = 0; 0%) (*p* = 0.0001) (Table 3).

**Table 3:** The status of rate control with diltiazem in the presence of polymorphism in *2, *3, *4 and *10 alleles and their statistical association

		Adequate rate control after one or two doses of diltiazem (%)	Inadequate rate control after one or two doses of diltiazem (%)	Total (%)	
*2	wt/wt	40 (71.4)	16 (28.6)	56 (100)	p* = 0.003
	wt/*2	12 (38.7)	19 (61.3)	31 (100)	
*3	wt/wt	39 (63.9)	22 (36.1)	61 (100)	p* = 0.225
	wt/*3	13 (50)	13 (50)	26 (100)	
*4	wt/wt	47 (75.8)	15 (24.2)	62 (100)	p* = 0.0001
	wt/*4	5 (20)	20 (80)	25 (100)	
*10	wt/wt	52 (66.7)	26 (33.3)	78 (100)	p** = 0.0001
	wt/*10	0 (0.0)	9 (100)	9 (100)	

Bold values indicate *p* < 0.05 which is statistically significant

p* values derived from Pearson Chi-square test

p** values derived from Fisher's exact chi-square test

Another aspect deserving attention is that the study patients were characterized by *1/*2 (n = 8), *1/*3 (n = 16), *1/*4 (n = 7), *2/*3 (n = 3), *2/*4 (n = 11), *2/*10 (n = 9), and *3/*4 (n = 7) genotypes. Rate control was achieved with diltiazem in all the subjects with *1/*2 genotype. Also rate control was achieved in all subjects with *1/*1 (n = 26) genotype. Adequate retarding effect could not be achieved in all *3/*4 and *2/*10 carriers (Table 4).

**Table 4:** Numerical values of the patients with adequate and inadequate rate control through diltiazem by genotypes

Genetic polymorphism	Adequate rate control with diltiazem	Inadequate rate control with diltiazem (%)	Total (%)
*1/*2	8 (%100.0) 1st dose (0.25 mg/kg): 8 2nd dose (0.35 mg/kg): 0	0 (0.0)	8 (100.0)
*1/*3	11 (%68.8) 1st dose (0.25 mg/kg): 6 2nd dose (0.35 mg/kg): 5	5 (31.2)	16 (100.0)
*1/*4	3 (%42.9) 1st dose (0.25 mg/kg): 0 2nd dose (0.35 mg/kg): 3	4 (57.1)	7 (100.0)
*2/*3	2 (%66.7) 1st dose (0.25 mg/kg): 0 2nd dose (0.35 mg/kg): 2	1 (33.3)	3 (100.0)
*2/*4	2 (%18,2) 1st dose (0.25 mg/kg): 0 2nd dose (0.35 mg/kg): 2	9 (81.8)	11 (100.0)
*3/*4	0 (%0.0)	7 (100.0)	7 (100.0)
*2/*10	0 (%0.0)	9 (100.0)	9 (100.0)
Total	26 (%42.6)	35 (57.4)	61 (100.0)

## Discussion

The aim of our study is to evaluate the relationship between CYP2D6 gene polymorphism and the efficacy of diltiazem, which is used in the acute retarding treatment of patients who present to the emergency department with atrial fibrillation with rapid ventricular response. These results support the need to understand the distribution of genetic biomarkers related to drug metabolism for planning national public health strategies. It shows a wide variation in enzyme activity among individuals. There is a wide spectrum of CYP2D6 alleles among different ethnic groups. Thus, poor metabolisers (poor metaboliser; PM), intermediate metabolisers (intermediate metabolizer; IM); When individuals are grouped as extensive metabolizers (EM) and ultrarapid metabolizers (UM), different rates of distribution can be observed in different populations. In addition, we aimed to determine the distribution of these polymorphisms in the population by using a healthy control group in our study. Predetermining the high risk in the variant group with CYP2D6 genotype screening can help the clinician to develop new dosage and follow-up protocols and implement the most effective and reliable treatment plan.

Probing the relationship between diltiazem efficacy and the genetic polymorphism of CYP2D6 in the patients suffering from AF with RVR, this study suggests that wt/wt (*1/*1), assumed to be the normal allele, is the most significant group in which drug efficacy is achieved. In the presence of *2, *4 and *10 alleles, drug efficacy was established to be significantly lower than the normal allele. On the other hand, we did not observe a significant association between the *3 allele and drug efficacy. As far as genotyping is concerned, drug administration proved to be efficacious in all the *1/*2 genotype carriers, but no drug efficacy was noted in any of their *3/*4 and *2/*10 counterparts.

Bae et al. [7] elucidated the association between pharmacokinetic properties of metoclopramide and the CYP2D6 polymorphism and documented a one-and-half-times elevation in the plasma concentration of metoclopramide and a 37% decrease in its clearance among wt/*10 carriers. By contrast, an Indonesia-based study hypothesized that the genetic polymorphism of CYP2D6 did not act on metoclopramide and ondansetron metabolisms [8]. Further research provided the clinical evidence that no difference was established between the *1/*1 and *1/*10 transporters in nebivolol metabolism, and that the *10 allele did not act on its metabolism [9]. However, these findings were not corroborated by other published research which revealed that the plasma concentration of nebivolol elevated by 30% in homozygous *10 allele carriers relative to individuals without polymorphism, and that the *10 allele did act on nebivolol metabolism [10]. Another clinical report on the relationship between metoprolol metabolism and the *10 allele indicated that half-life of metoprolol tended to be more prolonged in homozygous *10 allele carriers than normal or heterozygous gene carriers [11]. As identified by previous research on tramadol, homozygous CYP2D6*10 carriers reported significantly higher VAS scores than normal or heterozygous gene carriers [12]. The half-life of tramadol was also established to be 49.3% higher in *2/*10 gene carriers compared to normal variant carriers [13]. As far as our findings are concerned, *10 allele was observed as heterozygous combined with *2 allele in all the patients (n = 9), and successful rate control could not be achieved with diltiazem in any of these patients. In addition, drug efficacy was significantly reduced relative to the normal allele in the presence of *10 allele (*p* = 0.0001).

A clinical trial addressing the changes in metoprolol metabolism in the presence of the *4 allele reported that heart rate and diastolic blood pressure values were lower in homozygous (*4/*4) gene carriers than *1/*1 carriers. It was also noted that these patients were bearing an increased risk of bradycardia. The same study underlined that the CYP2D6 genotype exerted no effect on heart rate in individuals under atenolol regimen [14]. Our findings revealed that the provision of rate control with diltiazem were significantly lower in the individuals with polymorphic *4 allele than in their non-polymorphic counterparts (*p* = 0.0001). On the other hand, no adequate rate control could be achieved in any individuals carrying the *3/*4 genotype. We failed to establish a clear association between the presence of the *3 allele and successful rate control (*p* = 0.225), thus we are attributing the lack of drug efficacy in the *3/*4 genotype carriers to the effect of the *4 allele.

Polymorphism in the *2 allele may inhibit the metabolism of bufuralol and dextromethorphan by 40% relative to the *1 allele [15]. A clinical report on nebivolol documented that the decrease in the intrinsic clearance of nebivolol in the presence of the *2 allele was less than that of propranolol and dextromethorphan. It was interpreted that this condition was induced by the substrate specificity, and that these findings could not be generalized to other drugs [16]. Furthermore, Wang et al. reported that the CYP2D6*2 variant significantly impaired the catalytic activity in comparison to the *1 allele [17]. When it comes to our study, the presence of the *2 allele seemed to be responsible for dramatically decreased rate control with diltiazem compared to the *1/*1 normal allele (*p* = 0.003). The fact that some medications are unaffected by genetic polymorphism might stem from the substrate specificity of the enzyme.

Another line of evidence suggests that a greater decrease in heart rate during diltiazem exposure was observable in the individuals with poor metabolizers than with normal metabolizers [18]. However, our findings revealed that heart rate was not reduced in the presence of polymorphism of the *4 and *10 alleles, which tended to inhibit enzyme activity. The underlying reasons for this may be the absence of homozygous genes *4 and *10, which can contribute to further decrease in enzyme activity, in our participants or the metabolizing of diltiazem by other enzymes such as esterase and CYP3A4.

### Limitation of the study

The limited population and being a single-centered study are among our research limitations. Since no homozygous polymorphism was detectable in *2, *3, *4 and *10 alleles, we are unable to offer an explanation as to how this condition would act on drug efficacy. Because this study is observational, it is unknown to what extent the cumulative doses administered would affect clinical efficacy. Moreover, no explanation could be offered concerning the phenotype-drug efficacy since duplications were not taken into consideration in our study.

## Conclusion

Diltiazem proves to be a potent drug in patients with *1/*1 genotype without genetic polymorphism and presenting with AF with RVR. The presence of *2, *4 and *10 variant alleles was observed to significantly compromise the drug efficacy relative to the normal allele. On the other hand, the *3 allele was found to bear no relation to the effect of diltiazem on achieving rate control. Knowledge of clinical genotyping will guide clinicians in delivering the most appropriate treatment for patients.

## Data Availability

The datasets used and/or analysed during the current study are available from the corresponding author on reasonable request.

## Abbreviations

AF: Atrial fibrillation
CYP2D6: Cytochrome P450 2D6
DNA: Deoxyribonucleic acid
ED: Emergency department
EDTA: Ethylenediamine tetra-acetic acid
EM: Extensive metabolizer
Hr: Hour
IM: Intermediate metabolizer
IV: Intravenous
Kg: Kilogram
MAP: Mean arterial pressure
Mg: Miligram
Min: Minute
Ml: Mililiter
NYHA: New York Heart Association
PCR: Polymerase chain reaction
PM: Poor metabolizer
RVR: Rapid ventricular response
SBP: Systolic blood pressure
UM: Ultrarapid metabolizer
VAS: Visual analog scale
Wt: Wild type
